# Supplementary figures and images for: A new dataset for measuring the performance of blood vessel segmentation methods under distribution shifts
Source: PLoS One. 2025 May 27;20(5):e0322048. doi: 10.1371/journal.pone.0322048 (PMC12112280; doi:10.1371/journal.pone.0322048)

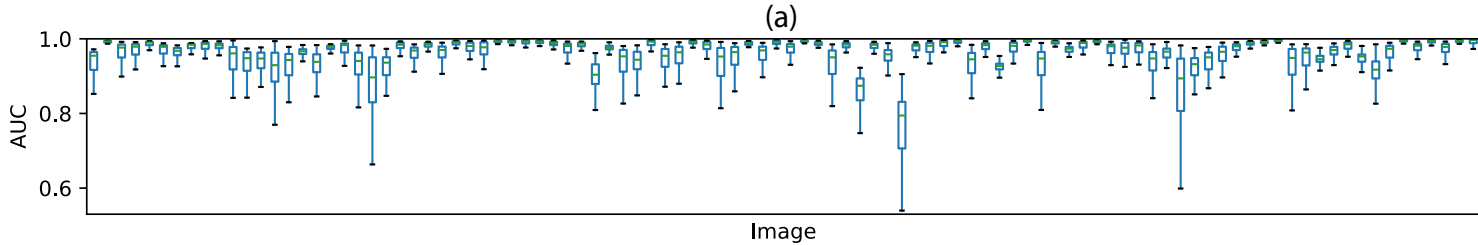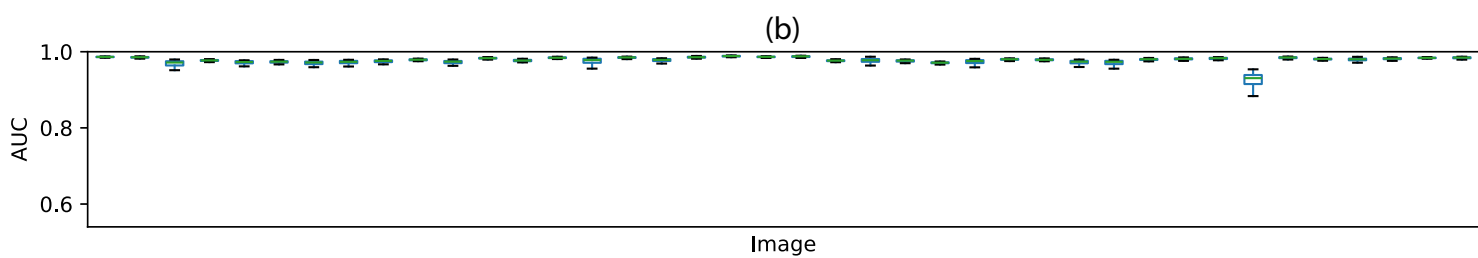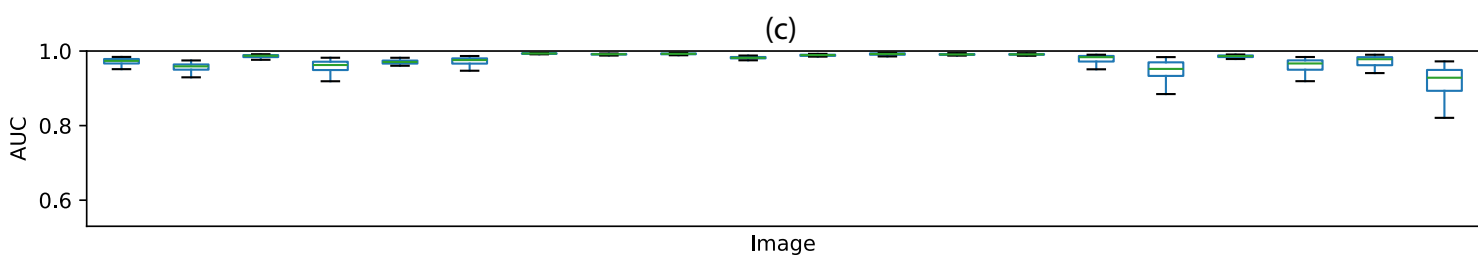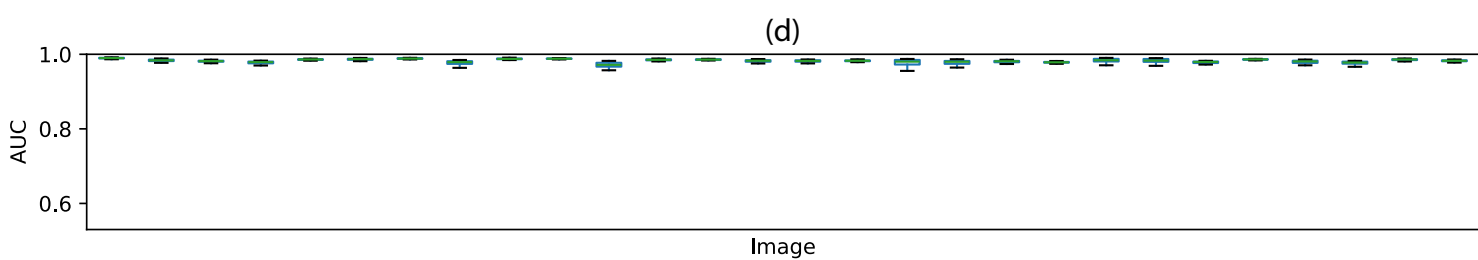

Supplement: S1 Fig — Each vertical box represents the distribution of the area under the ROC curve obtained for a sample across 100 training runs. The bottom and top of each box represent, respectively, the first (q1) and third (q3) quartiles of the data. The horizontal green line indicates the median and the whiskers indicate the range [q1−1.5(q3−q1),q3+1.5(q3−q1)]. The results are shown for the (a) VessMAP, (b) DRIVE, (c) STARE, and (d) CHASEDB1 datasets. (PDF) [file pone.0322048.s001.pdf]

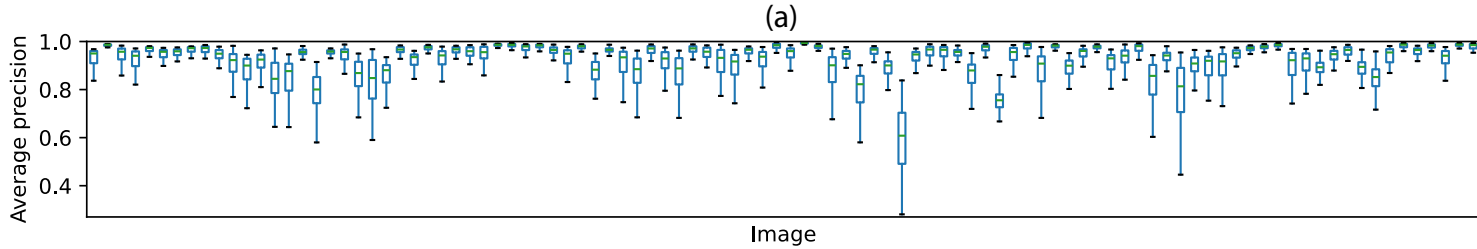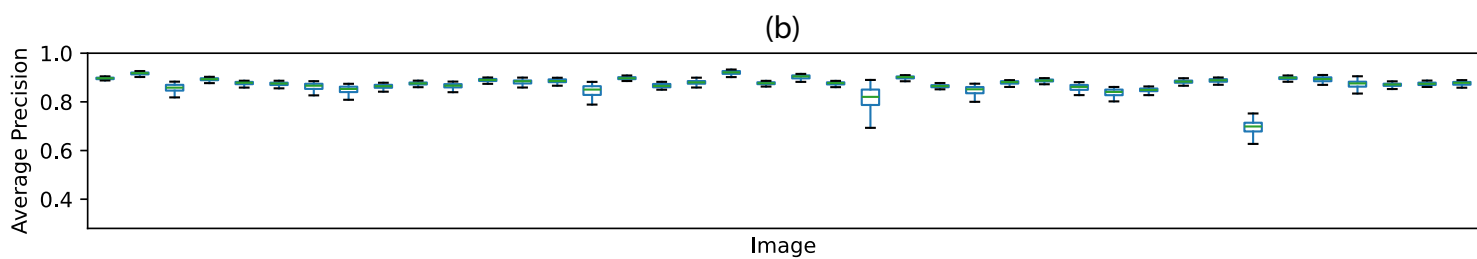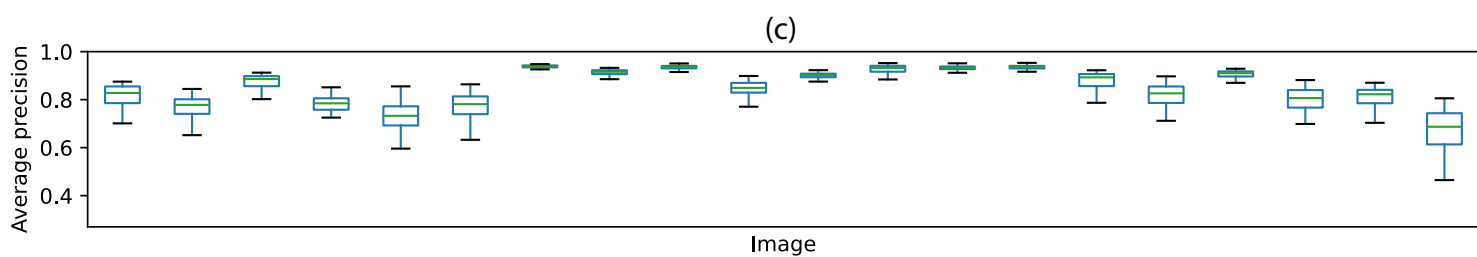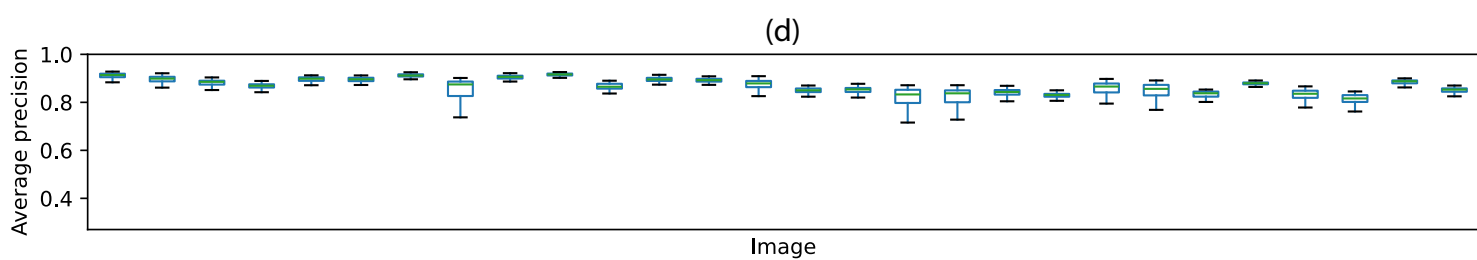

Supplement: S2 Fig — Each vertical box represents the average precision obtained for a sample across 100 training runs. The values were calculated as the average precision obtained when setting the decision threshold to each unique probability value. The bottom and top of each box represent, respectively, the first (q1) and third (q3) quartiles of the data. The horizontal green line indicates the median and the whiskers indicate the range [q1−1.5(q3−q1),q3+1.5(q3−q1)]. The results are shown for the (a) VessMAP, (b) DRIVE, (c) STARE, and (d) CHASEDB1 datasets. (PDF) [file pone.0322048.s002.pdf]
