# Supplementary material for: A new dataset for measuring the performance of blood vessel segmentation methods under distribution shifts
Source: PLoS One. 2025 May 27;20(5):e0322048. doi: 10.1371/journal.pone.0322048 (PMC12112280; doi:10.1371/journal.pone.0322048)
Supplement: Attachment — Submitted filename: response_letter_plos.pdf [file pone.0322048.s003.pdf]

Response to the Editor and reviewers

Editor comments

While preparing your manuscript, please make sure that you address the concerns raised by the reviewers related to the biases your proposed method may have introduced in the dataset generation method. Moreover, please include further statistical analysis metrics in your findings. The architectural details of the CNN used in this study need to be reported in detail for reproduction of these results. Furthermore, the choice of various parameters in the considered CNN have to be justified and compared against others in an ablation study to make sure no inductive bias has been introduced.

We have carefully revised the manuscript to address the concerns raised by the reviewers. Notably, to show that the results do not depend on a particular network architecture, another set of experiments using an established network architecture for blood vessel segmentation was included at the end of Section IV.B. This architecture was used on many recent works in the literature. For the custom network used elsewhere in the experiments, a figure was added to clearly describe the architecture. We thank the editor and the reviewers for the valuable comments, we believe they significantly improved the quality of the manuscript.

1.Please ensure that your manuscript meets PLOS ONE's style requirements, including those for file naming.
2. Please note that PLOS ONE has specific guidelines on code sharing for submissions in which author-generated code underpins the findings in the manuscript. In these cases, we expect all author-generated code to be made available without restrictions upon publication of the work.
3. Please state what role the funders took in the study.

We have carefully adapted the manuscript to follow PLOS ONE's style requirements. The final version of the code, after the review process, will be made available online. Also, we state that the funders had no role in study design, data collection and analysis, decision to publish, or preparation of the manuscript. As recommended, this statement was included in the cover letter.

Reviewer #1

1. Abstract

- Rewrite the abstract to:
- Highlight the practical applications of the proposed dataset and methods.
- Explicitly mention the novel aspects of the research.
- Include a brief summary of the key results.

We rewrote part of the abstract to describe more clearly the advantage of the VessMAP dataset over

other datasets from the literature. Also, some of the key results are now mentioned on the abstract. In addition, specific applications of the dataset are mentioned at the end of the abstract.

2. Introduction

- Expand the introduction to discuss how the methodology could be applicable to other areas, such as autonomous driving, aerial imaging, or manufacturing.
- Clearly articulate the specific gap in the literature that this research addresses.

We note that our work proposes a new blood vessel segmentation dataset. The dataset cannot be linked to autonomous driving, aerial imaging or manufacturing.

To explicitly state the main contributions of our work, a bulleted list was added at the end of the introduction.

3. Methodology

- Include a flowchart or diagram to visually represent the dataset creation process and evaluation framework.
- Provide more examples or illustrations of how the dataset was curated to ensure heterogeneity.

A new figure providing an overview of the analyses was included at the beginning of the methodology section (Figure 2, Section III). Also, additional details about the features used to ensure data diversity were provided in the last paragraph of Section III.B.1. Specifically, candidate features that are useful for characterizing vascular trees were implemented and calculated for the base dataset, and strongly correlated features were removed to produce a core set of features representing relevant properties of blood vessel samples.

4. Results

- Include a deeper analysis of the results, focusing on the strengths and weaknesses of the proposed approach.
- Compare the proposed method to additional state-of-the-art techniques to strengthen the validity of the conclusions.

We complemented the results to better show the main strength of the new dataset: the quantification of the generalization capability of segmentation methods trained on small sets of manually annotated samples. Specifically, the new experiments show that, for datasets with widespread use in the literature, networks trained on a small set of annotated images can trivially generalize to the whole dataset. In other words, images from these datasets are very similar to each other. On the other hand, it is not trivial to train a model on small sample sizes from the VessMAP dataset proposed in our work. This makes the dataset an excellent choice for developing new methods that need to be robust with the least

possible amount of manual annotation effort, such as in active learning and interactive segmentation tasks.

We also included a discussion regarding limitations of the dataset in paragraph 4 of the conclusion.

5. Practical Applications

- Add a section or paragraph that explores real-world scenarios where this research could have a significant impact (e.g., radiology diagnostics, AI-assisted surgery).
- Highlight the potential for this methodology to improve robustness in AI applications outside of healthcare.

A discussion regarding practical applications, with specific examples, was included in paragraphs 5 to 8 of the conclusion section.

6. Figures and Tables

- Add captions that provide more context and make the figures more standalone.
- Include qualitative results, such as segmented images from the dataset, to complement the quantitative metrics.

Figure captions were expanded to better describe the contents. Also, example segmentations were included (Figure 12).

7. Discussion

- Elaborate on the broader significance of the findings for machine learning and computer vision.
- Discuss potential limitations, such as computational requirements or biases in the dataset, and suggest ways to address them in future work.

Paragraphs 4 to 8 in the conclusion section were included to address practical applications, the broader significance and limitations of the dataset.

8. Conclusion

- Highlight how the dataset can serve as a benchmark for future research in segmentation under distribution shifts.
- Suggest specific avenues for extending the work, such as applying the methodology to larger or more diverse datasets.

As mentioned above, in the new version of the manuscript we discuss in detail practical applications of the dataset. The discussion is in paragraphs 5 to 8 of the conclusion.

9. Language and Readability

- Revise for grammatical correctness and conciseness.

- Use simpler language for technical terms to improve accessibility for readers outside the immediate field.

As suggested, we revised the grammar and spelling of the whole text and simplified it for readers of different expertise.

10. References

- Add citations from the latest studies (post-2021) to strengthen the relevance and contextualization of the research.

As suggested, we updated the references of the manuscript.

Reviewer #2

1. In the abstract, the authors should mention the achieved results and the model's improvement over existing approaches.

We rewrote the second part of the abstract. We now explicitly mention the main advantage of the VessMAP dataset over previous datasets and also specify some of the achieved performance scores when training a neural network using the dataset.

Also, we note that the main focus of the manuscript is the VessMAP dataset. The methodology used to uniformly sample relevant images to create the dataset, as well as the CNN model used to demonstrate the potential of the dataset, are not being proposed as novel. We changed the abstract, introduction and conclusion sections to better highlight the main contributions of the study.

2. In the related work section, various old papers are added. I suggested adding the recent relevant literature from 2022- 2024 to provide an updated overview to the readers.

Many datasets that are commonly employed in the literature are associated with relatively old papers. Still, we updated our review with recently published relevant datasets. Regarding other related concepts, we added a new paragraph (first paragraph of section IV.B) with examples of general applications of deep learning (as suggested in your fifth comment). In this paragraph, we took care to describe the most recent bibliography. In the conclusion section, recent references to large-scale biomedical datasets were also included on the last paragraph.

3. The paper's main contributions of the paper should be mentioned in points at the end of the introduction section.

As suggested, we listed the paper's main contributions at the end of the introduction section.

4. I did not find any table representing the results of the CNN model.

We included Table II with the training and validation losses and also with the δ values of our experiments with the VessMAP splits generated from the image features. Also, the results of the additional experiments included in Section IV.B are presented in Tables III and IV.

5. In the deep learning section, the authors are advised to add necessary introduction related to deep learning and its importance by citing the recent deep learning models such as iAFPs-Mv-BiTCN, AIPs-DeepEnC-GA, DeepAVP-TPPred, PAtbP-EnC, Deepstacked-AVPs, and AIPs-SnTCN for the reader's concern to provide a broader overview.

Relevant deep learning concepts and applications ware included in the first paragraph of Section IV.B.

6. An up to date comparison of the proposed model with existing state of the art models will be necessary.

We expanded the results section to include a more comprehensive comparison with datasets that are commonly employed in the literature. We show that, for many popular datasets, training sets containing just four samples can already provide a good performance for neural networks, and that the performance varies little according to the samples used for training. In contrast, Dice scores from 0.59 to 0.85 can be obtained depending on the VessMAP samples used for training. Thus, the training samples can be crucial for the performance. This makes the dataset an excellent choice for developing new active learning and interactive segmentation methods, where good and reliable performance must be obtained by annotating just a few carefully selected samples.

7. What should be the future directions of the proposed model

In order to suggest more specific applications, we included official training splits of the dataset leading to highly distinct inference performance. The splits are indicated in Table IV. In addition, at the end of the conclusion section we now discuss in more detail how the dataset and the splits can be used to develop new active learning and interactive segmentation methods.

Reviewer #3

The manuscript titled "A New Dataset for Measuring the Performance of Blood Vessel Segmentation Methods Under Distribution Shifts" introduces the VessMAP dataset, a highly heterogeneous collection of annotated fluorescence microscopy images. The dataset aims to improve the evaluation of segmentation algorithms, particularly under challenging conditions like distribution shifts.

1. The sampling methodology ensures diversity, but how does it account for potential biases introduced by the original dataset's composition? Could the over-representation of certain conditions affect

downstream analyses?

Indeed, this is an important concern that was not addressed in the text. Biomedical images may differ regarding the underlying condition, equipment used, tissue preparation protocol and imaging parameters. The VessMAP dataset can be used for testing the robustness of models when the samples have distinct characteristics (contrast, vessel density, etc). These characteristics were not linked to specific properties of the underlying condition or acquisition process of the samples in the base dataset since they strongly depend on specific applications. Thus, obtaining good performance on the VessMAP dataset is important, but not sufficient, to conclude that the model is not biased on downstream tasks. We included this discussion in paragraph 4 of the conclusion section.

2. You mention dual annotations for 20 samples. What metrics (e.g., Cohen's kappa, Dice similarity) were used to assess inter-annotator variability, and how do these findings support the reliability of your annotations?

The two annotators had a Dice similarity score of 0.8780 and a clDice similarity score of 0.9556. The Dice similarity score lies within the expected range of our neural network experiments – as stated in Section IV.B, our typical validation Dice values were between 0.82 and 0.89. Most disagreements between annotators are due to differences in the delineation of vessel borders, resulting in mildly different vessel caliber between annotators. The high clDice obtained emphasizes that the two annotators had similar criteria regarding the topology of the annotated blood vessels, which we consider of utmost importance in pipelines of automatic vessel morphometry. As a comparison, the annotations of the DRIVE dataset's (one of the most used blood vessel segmentation datasets) test set have a Dice similarity of 0.7881 and a clDice of 0.7634.

3. The features used (e.g., contrast, noise) are domain-specific. Could these features inadvertently bias model evaluations? Have you explored alternative or additional features that might better generalize to unseen data?

For our data selection, we employed relevant features for characterizing the morphology of vascular trees. We did explore additional options for the features. However, we disregarded metrics having strong correlation for our final data selection. In the revised manuscript, we clarify this point in Section III.B.1. As mentioned above, obtaining good performance on the VessMAP dataset is not sufficient to conclude that the model is not biased on downstream tasks. Indeed, to apply our data selection methodology to a new type of data, features that are significant to the new data domain should be employed, and not necessarily the four features used for VessMAP.
